# Oral and Dental Complications of Radiotherapy for Head and Neck Cancer: Knowledge of Dental Practitioners in Saudi Arabia

**DOI:** 10.31557/APJCP.2021.22.7.2033

**Published:** 2021-07

**Authors:** Ahmed Shaher Alqahtani, Yousef Alshamrani, Yaser Alhazmi, Esam Halboub

**Affiliations:** *Department of Oral and Maxillofacial Surgery and Diagnostics Sciences, College of Dentistry, Jazan University, Saudi Arabia. *

**Keywords:** Head and neck cancer, radiotherapy, knowledge, complication, dentists, oral and dental assessment

## Abstract

**Objectives:**

Toxicities of the oral soft and hard tissues due to the radiotherapy of the head and neck cancer can potentially lead to interruptions of cancer treatment and/or dose reduction, resulting in poorer outcomes. The aim of this study was to assess the knowledge of dental practitioners in Saudi Arabia about oral and dental assessment for and complications of radiotherapy of head and neck cancer.

**Materials and Methods:**

An online, already validated, self-administered questionnaire was sent via an online link through WhatsApp groups and other Social Media platforms to reach out to the majority of targeted samples (dental practitioners working in Saudi Arabia). Responses were statistically described and analyzed based on the different grouping factors: gender, specialty, working sector, region of work and experience.

**Results:**

There were 370 respondents, 257 (69.5%) of them were males. Most of the respondents were general dental practitioners [144 (38.9%)], The percentages of the correct answers range from as low as 26.2 to as high as 97%. The per cent of correct answers by the respondents in 18 out of 31 questions was above 75%. Females, dental specialists (specifically prosthodontics), working in public sectors and in the central and western regions of Saudi Arabia were associated with higher levels of knowledge.

**Conclusion:**

Our results show highly variable knowledge of dental practitioners on oral and dental assessment for, and complications and management of radiotherapy to the head and neck area; that knowledge seems to fluctuate considerably with gender, experience, work sector and specialty.

## Introduction

Head and neck cancer (HNC) is an umbrella term refers to a group of cancers developing in the oral cavity, pharynx, larynx, paranasal sinuses, nasal cavity, salivary glands, or lymph nodes of the head and neck; it is considered the ninth most common cancer worldwide (Amin et al., 2017). Oral cancer accounts for about 26% among all the head and neck cancers recorded annually in the Kingdom of Saudi Arabia (KSA), and greater number of them is detected at advanced stage. Oral cancer in KSA is strongly linked to the frequent use of smokeless tobacco (Shammah) (Quadri et al., 2019; Patil et al., 2019) and to the chewing of qat (Quadri et al., 2015), an evergreen shrub chewed for it’s an amphetamine-like effects (Al-Hebshi and Skaug, 2005). A large number of oral cancer cases are documented from the southwestern region of KSA especially in Jazan province and are invariably related to the consumption of Shammah(Quadri et al., 2019; Patil et al., 2019; Quadri et al., 2015).

Surgery, radiotherapy, or chemotherapy are commonly employed, either individually or in combination, for treatment of HNC. Radiotherapy to oropharyngeal region may induce squamous epithelium destruction, inhibit proliferation of transit cells, and absence of cell regeneration leads to acute mucositis. Also, high-dose radiation to tooth-supporting bone results in hypoxia and reduces the vascular supply to the bone and soft tissues, causing fibrosis and vascular thromboses (Shih et al., 2003). Given the treatment-related toxicities to the soft and hard tissues of the oral region, cancer treatment might be interrupted, or dose might be reduced, resulting in poorer outcomes such as increased morbidity and possibly decreased survival (Jasmer et al., 2020). 

Oral and dental complications include mucositis, infections, pain, salivary gland dysfunction, taste change, dysphagia, trismus, and soft and hard tissue necrosis (Martinez et al., 2020; Bonar-Alvarez et al., 2020; Brook, 2020). Therefore, dental assessment and management of HNC patient pre and post cancer treatment is one of the cornerstones in the comprehensive care approach (Bacher et al., 2020; Colloc et al., 2020; Moore et al., 2020). The main aim of doing so is to eliminate or at least reduce the risk of the above mentioned complications. Thus, it is essential that all health professionals including dentists need to be aware of and knowledgeable about the prevention, diagnosis, and management of oral complications of radiotherapy in order to ultimately minimize the impact of these complications on the patient’s life (Cho and Kumar, 2019; McCaul, 2012). In this context, a few studies have assessed this issue and revealed variable knowledge among dental practitioners/students (Suhaimi, 2017; Dewan et al., 2014; Mainali et al., 2011; Guneri et al., 2008). No single study has been conducted to investigate the knowledge and awareness among dentists in Saudi Arabia towards oral and dental assessment and management of head and neck cancer patients pre and post-radiotherapy, and hence this study sought to do so.

## Materials and Methods

*Methodology*

This study is of cross-sectional, questionnaire-based study design. The population framework was dental practitioners working currently in Saudi Arabia. The questionnaire’s items were taken from previously validated questionnaires used in the same context (Mainali et al., 2011; Guneri et al., 2008). For more confirmation, the questionnaire was administered to three experts in oral medicine and pathology, who had a clear understanding of the pre and post radiotherapy oral complications and proper managements. Their feedbacks were minor, and the questionnaire was corrected accordingly. Then, the questionnaire was administered to 18 dentists from different dental specialties and asked whether they had difficulty in understanding any questions. Their feedbacks showed that the questionnaire was appropriate and easy to understand; minor corrections were made at this stage. 

The questionnaire included 20-questions pertaining to the knowledge on oral/dental assessment for and management of HNC patients pre- and post-radiotherapy. The first 5 items of the questionnaire were about the participants’ demographic information (gender, specialty, working field, working experience and region of work in KSA). Of the other 15 items, 7 were directed to the oral/dental assessment and information prior to radiotherapy. The remaining 8 items are related to oral complications of radiotherapy and their management protocols. The stems and alternatives (answers) of all items are listed in Table 2.

To confirm nationwide distribution, the authors were keen to target as many as possible dental practitioners with different background (general dentists, specialists, consultants, academics) at different working fields (Private clinic, public hospital, health care center and universities) and in the five regions of the KSA (Central, Western, Eastern, Northern and Southern). To do so, the questionnaire was administered to the potential participants using Google form via an online link through WhatsApp groups and other Social Media platforms depending on friends in the different regions of KSA. Furthermore, five reminders were sent through these channels aiming to increase the response rate. Responses to the questionnaire were sensitive to the Internet Protocol (IP) Address assuring no duplicated responses. The time frame during which the questionnaire was circulated was between 26^th^ May and 18^th^ June 2020.The study protocol was approved in advance and Ethical Clearance was obtained from College of Dentistry, Jazan University. The research was self-funded and there are no conflicts of interest to be reported.

*Statistical analysis*

Data were obtained in “excel” format and exported into SPSS program, Version 21 (Armonk, NY: IBM Corp) for further analyses. As all data were qualitative, they were presented as frequencies and corresponding percentages. All variables were described for the whole sample, and analyzed by different grouping factors (gender, specialty, work sector, year of experience, and region of the work) using Chi square/Fisher exact tests as appropriate. For questions with multiple correct/possible choices (“Mark all that apply”), each choice was statistically dealt with as a separate variable with “positive/yes” answer when marked and “negative/no” answer when not marked. A P value of less than 0.05 was considered significant.

## Results

There were 370 respondents, 257 (69.5%) of them were males. Most of the respondents were general dental practitioners [144 (38.9%)], followed by oral surgery/medicine/pathology specialists [57 (15.4%)], endodontists [34 (9.2%)], and periodontists [32 (8.6%)]; the other specialties represented up to 28%. Most of the respondents reported working in universities [234 (63.2%)], and half of them had less than 5 years of experience [183 (49.5%)]. More than 63% of the respondents worked in the central and western region of the kingdom (Table 1).

(Table 2) presents the responses of the whole sample to the included knowledge questions. Worthy to mention that (Table 2) presents the whole questions’ stems with their choices and can be referred to while reading the subsequent tables. The bold responses indicate correct/possible answers. The percentages of the correct answers range from as low as 26.2 for “The oral cavity is often subject to complications from radiotherapy because: all foods aggravate weakened tissues” to as high as 97% for “As a dentist, do you think that there is any necessity for oral/dental assessment before radiotherapy for head and neck cancer patients?” The per cent of correct answers by the respondents in 18 questions out of 31 was above 75%. 

Gender-wise comparisons showed higher knowledge in favor of females (Table 3). For example, higher proportion of females than males marked dental caries (83.2% vs. 66.4) and osteoradionecrosis (92.9% vs. 84.8%) as potential complications of head and neck radiotherapy that must be discussed with the patients ahead of the commencing the treatment. Meanwhile lower proportion of females than males (12.4% vs. 23.3%) marked “The tissue in the oral cavity thickens” as a cause of complications of head and neck radiotherapy. One exception was that more females than males (85% vs. 75.1%) thought that “Alcohol containing mouthwash” should be recommended by the dentists before head and neck radiotherapy.

(Table 4) presents the distribution of responses by dental specialty. Obviously, specialty variable showed the vast majority of differences. The general dentists showed lower knowledge compared to the other specialties. Surprisingly, oral surgery/medicine/pathology, prosthodontists and periodontists showed higher knowledge in most of the included questions. For example, 36.5%, 37.5% and 56% of oral surgery/medicine/pathology, prosthodontists and periodontists, respectively, reported that oral complications may lead to lowering the dosages and possibly discontinuing cancer treatment in comparison to 25%, 17%, and 25.3% of general dentists, endodontists and other specialties.

The responses based on work sector are presented in (Table 5). Dentists or dental specialists working in universities or public hospitals revealed higher knowledge compared to those working in the private clinics and health care centers. For example, 89.9% and 87.4% of those working in public hospitals and universities, respectively, reported that patients undergoing cancer treatment may experience alterations in taste perception compared to 69.2% and 77.8% of those working in health care center and private clinic/center, respectively.

Few differences were reported based on the year of experience as shown in (Table 6), with no constant association with specific duration of dental profession, although those who worked less than 5 years showed somewhat lower knowledge. For example, 23% of whose experience was less than 5 years reported that the tissue in the oral cavity thickens with radiotherapy in comparison to 21.7% and 9% of whose experience was between 5-10 years and more than 10 years, respectively.

With regard to the region of work, dentist and dental specialists working in the central and western regions showed the highest levels of knowledge in most of the included questions, while those working in the northern and to less extent those working in the eastern regions revealed the lowest levels of knowledge (Table 7). For example, 94.8% and 96.8% of those working in the central and western regions, respectively, suggested extraction of teeth with poor prognosis ahead of radiotherapy in comparison to 79.2%, 80.8% and 80.8% of those working in the eastern, northern and southern regions, respectively, who reported the same.

**Table 1:** Demographic Data of the Sample

Variable	Categories	n (%)
Gender	Males	257 (69.5)
	Females	113 (30.5)
Specialty	General Dentists	144 (38.9)
	Oral surgery/Medicine/Pathology	57 (15.4)
	Endodontist	34 (9.2)
	Periodontist	32 (8.6)
	Prosthodontist	16 (4.3)
	Others	87 (23.5)
Working field	Health care center	39 (10.5)
	Private clinic	18 (4.9)
	Public hospital	79 (21.4)
	University	234 (63.2)
Working experience as a dentist	Less than 5 years	183 (49.5)
	5-10 years	120 (32.4)
	More than 10 Years	67 (18.1)
Working Region	Eastern	24 (6.4)
	Western	63 (17)
	Northern	48 (13)
	Southern	120 (32.4)
	Central	115 (31.1)

**Table 2:** Responses* of the Whole Sample

Questions	Choices	N (%)
Q6. As a dentist, do you think that there is any necessity for oral/dental assessment before radiotherapy for head and neck cancer patients?	**Yes**	**359 (97)**
	No	2 (0.5)
	I don’t know	9 (2.4)
Q7. The ideal time to do a comprehensive oral evaluation for head and neck cancer patients:	**After a cancer diagnosis and before radiotherapy**	**342 (92.4)**
	After finishing radiotherapy	14 (3.8)
	During radiotherapy	5 (1.4)
	Only as needed	9 (2.4)
Q8. Before radiotherapy for head and neck cancer patient, oral/dental assessment and management should include: "Mark all that apply"	**Thorough hard and soft tissue examination **	**344 (93)**
	**Appropriate radiographs such as full mouth x-ray and panorama **	**295 (79.7)**
	Extraction of deeply impacted teeth without pathology	115 (31.1)
	**Fluoride application**	**200 (54.1)**
	**Extraction of teeth with poor prognosis**	**322 (87)**
Q9. Which oral problems associated with radiotherapy need to be discussed with head and neck cancer patients before radiotherapy? "Mark all that apply"	**Oral Mucositis **	**279 (75.4)**
	Hypersalivation	46 (12.4)
	**Loss or change of taste sensation **	**302 (81.6)**
	**Oral candidiasis**	**265 (71.6)**
	**Difficulty in mouth opening**	**195 (52.7)**
	**Dental caries**	**265 (71.6)**
	**Osteoradionecrosis**	**323 (87.3)**
Q10. What should the dentist recommend for head and neck cancer patients before radiotherapy? "Mark all that apply"	**Alcohol-free antiseptics**	**220 (59.5)**
	Hard toothbrush	22 (5.9)
	**Fluoride toothpaste**	**304 (82.2)**
	Alcohol containing mouthwash	53 (14.3)
	**Salivary substitute**	**289 (78.1)**
	Sugar-containing food and drinks	18 (4.9)
Q11. The ideal time to begin radiotherapy after oral surgery such as teeth extraction:	2-3 days	7 (1.9)
	4 -7 days	20 (4.5)
	After a week	48 (13)
	**After two weeks**	**267 (72.2)**
	As soon as possible	28 (7.6)
Q12. Is oral prophylaxis (teeth cleaning) recommended before radiotherapy?	**Yes**	**312 (84.3)**
	No	40 (10.8)
	I don’t know	18 (4.9)
Q13. How often head and neck cancer patients need to follow up with a dentist post-radiotherapy?	**Every 3-4 months**	**296 (80)**
	Once a year	12 (3.2)
	Twice a year	53 (14.3)
	Only as needed	9 (2.4)
Q14. How does the dentist usually handle post-radiation oral complications? "Mark all that apply"	**Dentist treats by him/herself**	**122 (33)**
	**Refer to a dental specialist**	**319 (86.2)**
	Refer to otolaryngologist	91 (24.6)
	No need for any treatment	7 (1.9)
Q15. The oral cavity is often subject to complications from radiotherapy because: ”Mark all that apply”	**All foods aggravate weakens tissues**	**97 (26.2)**
	Many patients experience hypersalivation	28 (7.6)
	The tissue in the oral cavity thickens	74 (20)
	**Soft tissues in the mouth become easily damaged and infected**	**342 (92.4)**
Q16. Which of the following statements about the oral complications of head and neck cancer radiotherapy are correct? ”Mark all that apply”	**Oral mucositis can increase the risk of oral pain and systemic infection**	**300 (81.1)**
	**Oral complications may lead to lowering the dosages and possibly discontinuing cancer treatment**	**106 (28.6)**
	High dosages of radiation cannot affect dental or skeletal development in children	21 (5.7)
	**Patients undergoing cancer treatment may experience alterations in taste perception**	**316 (85.4)**
	Choices	**N (%)**
	There is a change in the flow rate for saliva	**344 (93)**
	There is a change in the composition of saliva	**151 (40.8)**
	The ability to taste is impaired	**74 (20)**
	Daily self-application of topical fluoride	**28 (7.6)**
	Sip water	**265 (71.6)**
	Chew sugarless gum	**342 (87.6)**
	Eat spicy foods to stimulate the salivary glands	**26 (6)**
	Use liquid to soften foods	**222 (60)**
	Mouthrinse containing alcohol	**75 (20.3)**
	Lip balm	**134 (36.2)**
	Topical anesthetics	**295 (79.7)**
	Chewing ice	**95 (25.7)**
	When the patient experiences mouth pain	**57 (15.4)**
	When the patient has difficulty swallowing	**42 (11.4)**
	When the patient has an oral infection	**66 (17.5)**
	Never	**270 (73)**

*, Yes responses are presented for each choice regarding “Mark all that apply” questions; Bolded responses represent the correct/possible answers.

**Table 3:** Responses* by Gender (Only Significant Associations are Presented)

Questions^†^	Choices	Gender		P value^Ŧ^
		Males n (%)	Females n (%)	
Q9	Extraction of teeth with poor prognosis	215 (83.3)	108 (95.6)	0.001
	Loss or change of taste sensation	201 (78.2)	101 (89.4)	0.013
	Dental caries	171 (66.5)	94 (83.2)	0.001
	Osteoradionecrosis	218 (84.8)	105 (92.9)	0.041
Q10	Fluoride toothpaste	203 (79)	101 (89.4)	0.018
	Alcohol containing mouthwash	193 (75.1)	96 (85)	0.04
Q13	Every 3-4 months	213 (82.9)	83 (73.5)	0.028
	Once a year	10 (3.9)	2 (1.8)	
	Twice a year	28 (10.9)	25 (22.1)	
	Only as needed	6 (2.3)	3 (2.7)	
Q15	All foods aggravate weakened tissues	78 (30.4)	19 (16.8)	0.007
	The tissue in the oral cavity thickens	60 (23.3)	14 (12.4)	0.016
	Soft tissues in the mouth become easily damaged and infected	231 (89.9)	111 (98.2)	0.005
Q16	Oral mucositis can increase the risk of oral pain and systemic infection	201 (78.2)	99 (87.6)	0.043
	Oral complications may lead to lowering the dosages and possibly discontinuing cancer treatment	82 (31.9)	24 (21.2)	0.045

^*^, Yes responses are presented for each choice regarding “Mark all that apply” questions; †, The corresponding questions are presented in Table 2. ^Ŧ^, Chi square/Fisher exact tests as appropriate.

**Table 4:** Responses* by Specialty (Only Significant Associations are Ppresented)

Questions^†^	Choices	Specialty						P value^Ŧ^
		GD	OSMP	Endo	Perio	Pros	Others	
Q8	Appropriate radiographs such as full mouth x-ray and panorama	108 (75)	49 (86)	30 (88.2)	29 (90.6)	16 (100)	63 (72.4)	0.014
	Extraction of deeply impacted teeth without pathology	40 (27.8)	27 (47.4)	7 (20.6)	13 (40.6)	4 (25)	24 (27.6)	0.038
	Fluoride application	60 (41.7)	40 (70.2)	18 (52.9)	15 (46.9)	13 (81.3)	54 (62.1)	<0.001
	Extraction of teeth with poor prognosis	116 (80.6)	56 (98.2)	26 (76.5)	31 (96.9)	15 (93.8)	78 (89.7)	0.002
Q9	Oral Mucositis	94 (65.3)	52 (91.2)	26 (76.5)	28 (87.5)	16 (100)	63 (72.4)	<0.001
	Loss or change of taste sensation	116 (80.6)	51 (89.5)	22 (64.6)	28 (87.5)	16 (100)	69 (79.3)	0.018
	Oral candidiasis	83 (57.6)	51 (89.5)	27 (79.4)	26 (81.3)	13 (81.3)	65 (74.7)	<0.001
	Difficulty in mouth opening	59 (41)	49 (86)	11 (32.4)	20 (62.5)	12 (75)	44 (50.6)	<0.001
	Dental caries	82 (56.9)	47 (82.5)	28 (82.4)	25 (78.1)	15 (93.8)	68 (78.2)	<0.001
	Osteoradionecrosis	82 (56.9)	47 (82.5)	28 (82.4)	28 (78.1)	15 (93.8)	78 (89.7)	<0.001
Q10	Alcohol-free antiseptics	75 (52.1)	30 (52.6)	19 (55.9)	22 (68.8)	13 (81.3)	61 (70.1)	0.023
	Hard toothbrush	10 (6.9)	1 (1.8)	6 (17.6)	0 (0)	0 (0)	5 (5.7)	0.022
	Fluoride toothpaste	106 (73.6)	55 (96.5)	28 (82.4)	26 (81.3)	15 (93.8)	74 (85.1)	0.004
Q12	Yes	110 (76.4)	52 (91.2)	32 (94.1)	32 (100)	14 (87.5)	72 (82.8)	0.004
	No	8 (5.6)	4 (7)	0 (0)	0 (0)	2 (12.5)	4 (4.6)	
	I don’t know	26 (18.1)	1 (1.8)	2 (5.9)	0 (0)	0 (0)	11 (12.6)	
Q14	Refer to a dental specialist	115 (79.9)	51 (91.2)	33 (97.1)	30 (93.8)	16 (100)	73 (83.9)	0.016
	Refer to otolaryngologist	51 (35.4)	11 (19.3)	3 (8.8)	9 (28.1)	2 (12.5)	15 (17.2)	0.002
Q15	The tissue in the oral cavity thickens	42 (29.2)	11 (19.3)	2 (5.9)	5 (15.6)	6 (37.5)	8 (9.2)	0.001
Q16	Oral complications may lead to lowering the dosages and possibly discontinuing cancer treatment	36 (25)	21 (36.5)	6 (17.6)	12 (37.5)	9 (56.3)	22 (25.3)	0.028
Q17	There is a change in the flow rate for saliva	140 (97.2)	52 (91.2)	32 (94.1)	31 (96.9)	13 (81.3)	76 (87.4)	0.029
Q18	Eat spicy foods to stimulate the salivary glands	9 (6.3)	3 (5.3)	0 (0)	0 (0)	2 (12.5)	12 (13.8)	0.033
Q19	Topical anesthetics	124 (86.1)	46 (80.7)	20 (58.5)	28 (87.5)	11 (68.8)	66 (75.9)	0.007

^*^, Yes responses are presented for each choice regarding “Mark all that apply” questions; ^†^, The corresponding questions are presented in Table 2. ^Ŧ^, Chi square/Fisher exact tests as appropriate.

**Table 5:** Responses* by Work Sector (Only Significant Associations are Presented)

Questions^†^	Choices	Specialty				P value^Ŧ^
		Health care center	Private clinic/center	Public hospital	University	
Q6	Yes	36 (92.3)	17 (94.4)	78 (98.7)	228 (97)	0.047
	No	0 (0)	1 (5.6)	0 (0)	1 (0.4)	
	I don’t know	3 (7.7)	0 (0)	1 (1.3)	5 (2.1)	
Q7	After a cancer diagnosis and before radiotherapy	36 (92.3)	13 (72.2)	75 (94.9)	218 (93.2)	0.004
	After finishing radiotherapy	1 (2.9)	4 (22.2)	3 (3.8)	6 (2.6)	
	During radiotherapy	2 (5.1)	1 (5.6)	0 (0)	2 (0.9)	
	Only as needed	0 (0)	0 (0)	1 (1.3)	8 (3.4)	
Q8	Fluoride application	12 (30.8)	8 (44.4)	45 (57)	135 (57.7)	0.013
Q9	Oral Mucositis	23 (59)	10 (55.6)	62 (78.5)	184 (78.6)	0.011
	Difficulty in mouth opening	17 (43.6)	8 (44.4)	28 (35.4)	142 (60.7)	<0.001
	Osteoradionecrosis	29 (74.4)	13 (72.2)	72 (91.1)	209 (89.3)	0.011
Q10	Hard toothbrush	5 (12.5)	4 (22.2)	2 (2.5)	11 (4.7)	0.005
Q12	Yes	28 (71.8)	14 (77.8)	69 (87.3)	201 (85.9)	0.011
	No	0 (0)	1 (5.6)	4 (5.1)	13 (5.6)	
	I don’t know	11 (28.2)	3 (16.7)	6 (7.6)	20 (8.5)	
Q14	Refer to a dental specialist	31 (79.5)	11 (61.1)	15 (81)	213 (91)	0.001
	No need for any treatment	0 (0)	4 (22.2)	0 (0)	3 (1.3)	<0.001
Q15	The tissue in the oral cavity thickens	12 (30.8)	7 (38.9)	10 (12.7)	45 (19.2)	0.022
Q16	High dosages of radiation cannot affect dental or skeletal development in children	1 (2.6)	3 (16.7)	1 (1.3)	16 (6.3)	0.044
	Patients undergoing cancer treatment may experience alterations in taste perception	27 (69.2)	14 (77.8)	71 (89.9)	204 (87.4)	0.012

^*^, Yes responses are presented for each choice regarding “Mark all that apply” questions; ^†^, The corresponding questions are presented in Table 2. ^Ŧ^, Chi square/Fisher exact tests as appropriate.

**Table 6:** Responses* by Experience (Only Significant Associations are Presented)

Questions^†^	Choices	Experience			P value^Ŧ^
		< 5 years	5-10 years	> 10 years	
Q8	Extraction of teeth with poor prognosis	161 (88)	109 (90.8)	52 (77.6)	0.031
Q9	Dental caries	120 (65.6)	93 (77.5)	52 (77.6)	0.038
	Osteoradionecrosis	163 (89.1)	108 (90)	52 (77.6)	0.032
Q10	Alcohol containing mouthwash	37 (20.2)	9 (7.5)	7 (10.4)	0.005
Q15	The tissue in the oral cavity thickens	46 (25.1)	42 (35)	9 (13.4)	0.005
	Many patients experience hypersalivation	20 (10.9)	4 (3.3)	4 (6)	0.046
	The tissue in the oral cavity thickens	42 (23)	26 (21.7)	6 (9)	0.045
Q19	Chewing ice	45 (24.6)	39 (32.5)	11 (16.4)	0.047

^*^, Yes responses are presented for each choice regarding “Mark all that apply” questions. ^†^, The corresponding questions are presented in Table 2. ^Ŧ^, Chi square/Fisher exact tests as appropriate.

**Table 7:** Responses* by Region of Work (Only Significant Associations are Presented)

Questions^†^	Choices	Region					P value^Ŧ^
		Central	Eastern	Northern	Southern	Western	
Q6	Yes	112 (97.4)	21 (87.5)	45 (93.6)	119 (99.2)	62 (98.4)	0.03
	No	0 (0)	1 (4.2)	0 (0)	1 (0.8)	0 (0)	
	I don’t know	3 (2.6)	2 (8.3)	3 (6.3)	0 (0)	1 (1.6)	
Q8	Fluoride application	76 (66.1)	14 (58.3)	16 (33.3)	56 (46.7)	38 960)	0.001
	Extraction of teeth with poor prognosis	109 (94.8)	19 (79.2)	97 (80.8)	97 (80.8)	61 (96.8)	<0.001
Q9	Oral Mucositis	97 (84.3)	13 (54.2)	26 (54.2)	90 (75)	53 (84.1)	<0.001
	Oral candidiasis	94 (81.7)	10 (41.7)	30 (62.5)	82 (68.3)	49 (77.8)	<0.001
	Difficulty in mouth opening	63 (54.8)	13 (54.2)	14 (29.2)	65 (54.2)	40 (63.5)	0.007
	Dental caries	90 (78.3)	13 (54.2)	27 (56.,3)	82 (68.3)	53 (84.1)	0.002
	Osteoradionecrosis	103 (89.6)	21 (87.5)	38 (79.2)	100 (83.3)	61 (96.8)	0.036
Q10	Alcohol-free antiseptics	62 (53.9)	7 (29.2)	26 (54.2)	83 (69.2)	42 (66.7)	0.002
	Hard toothbrush	2 (1.7)	1 (4.2)	6 (12.5)	12 (10)	1 (1.6)	0.012
	Fluoride toothpaste	106 (92.2)	17 (70.8)	33 (68.8)	92 (76.7)	56 (88.9)	0.001
	Salivary substitute	94 (81.7)	17 (70.8)	30 (62.5)	92 (76.7)	56 (88.9)	0.012
Q14	Refer to otolaryngologist	19 (16.5)	7 (29.2)	17 (37.5)	36 (30)	11 (17.5)	0.015
	No need for any treatment	1 (0.9)	0 (0)	4 (8.3)	2 (1.7)	0 (0)	0.017
Q15	All foods aggravate weakened tissues	19 (16.5)	5 (20.8)	12 (25)	43 (35.8)	18 (28.6)	0.018
	Many patients experience hypersalivation	1 (0.9)	2 (8.3)	9 (18.8)	11 (9.2)	5 (7.9)	0.003
	The tissue in the oral cavity thickens	13 (11.3)	4 (16.7)	15 (31.3)	26 (21.7)	16 (25.4)	0.029
	Soft tissues in the mouth become easily damaged and infected	114 (99.1)	20 (83.3)	42 (87.5)	104 (86.7)	62 (98.2)	0.001

^*^, Yes responses are presented for each choice regarding “Mark all that apply” questions. ^†^, The corresponding questions are presented in Table 2. ^Ŧ^, Chi square/Fisher exact tests as appropriate.

## Discussion

Oral and paraoral complications of radiotherapy to the head and neck area are well-documented, and so are the proposed guidelines for management that are continually updated (Kumar et al., 2018; Nekhlyudov et al., 2018; Nekhlyudov et al., 2017; Samim et al., 2016; Beech et al., 2014; Plemons et al., 2013; Hancock et al., 2003). However, little is known about the extent to which dental practitioners are aware and knowledgeable about these complications and their proper management. Hence, this study sought to assess the knowledge and practice of dental practitioners working in Saudi Arabia in this context. 

The results revealed astonishing knowledge in terms of the need for, and the proper timing of, dental/oral assessment for the cancer patients who are to undergo radiotherapy to the head and neck area (97% and 92%, respectively). Contrastingly, the results unveiled highly variable knowledge levels when it comes to the individual radiotherapy complications and their individual management procedures; the correct answers ranged from as low as 26% to as high as 93%. Somewhat similar knowledge levels, or even lower, were reported in a similar study targeted Turkish dentists (Guneri et al., 2008). The picture was worse concerning Turkish senior dental students; their knowledge levels ranged from 5.2 to 98.7 % (Alopz et al., 2013). Variable knowledge was reported too in the approach of practitioners in restorative dentistry (Dewan et al., 2014) and oncology (Mainali et al., 2011) in context of radiotherapy for head and neck cancer patients. It seems that this issue is neither covered properly in the educating institutes nor dealt seriously by the dental/oncologic relevant authorities. Indeed, a recent study concluded that only 48.6% New Zealand and 2.5% Malaysian dentists followed formal guidelines or protocols for dental assessment of head and neck cancer patients, and hence recommended developing/[activating the current] clinical guidelines and generalizing them to all relevant practitioners in order to support effective medical/dental treatment and management strategies for this vulnerable population (Suhaimi, 2017).

It was surprising that 31% of the respondents reported that oral/dental assessment and management should include “Extraction of deeply impacted teeth without pathology.” It is an axiom that the intervention should not be performed on teeth that don’t represent sources of infection irrespective of the systemic conditions the patients have, or the treatment modalities planned. Basically, one of the guidelines for this class of patients is that “All healthy teeth as well as deeply impacted teeth without pathology are left in situ” (Beech et al., 2014). 

It was also surprising that 81% that “oral mucositis can increase the risk of oral pain and systemic infection.” Although oral mucositis represents a real problem associated with poor oral functions including difficulties in eating and swallowing which ultimately lead to poor quality of life (Martins et al., 2020; Morais et al., 2020), it is not linked directly with systemic infections. One of the low-scored knowledge items was that 71% of the respondents didn’t though that “oral complications may lead to lowering the dosages and possibly discontinuing cancer treatment”. In fact, the more sever the oral complications the more urgent the need to suspend the radiotherapy (Morais et al., 2020; Agarwal et al., 2012). 

More than 14% of the respondents reported that “alcohol-based mouthwashes” can be recommended for head and neck cancer patients before radiotherapy and 20% reported that these mouthwashes can be prescribed to control pain during or after radiotherapy. One of the main guidelines in management of the head and neck patients who are either to undergo or currently under radiotherapy, however, is to use alcohol-free mouthwashes and/or topical preparations (Kumar et al., 2018; Nekhlyudov et al., 2018; Nekhlyudov et al., 2017; Suhaimi, 2017; Samim et al., 2016).

Although no differences by gender, age, experience and specialty were reported in Güneri study in Turkey, our study concluded contradicting results; female dental practitioners revealed better knowledge in many of the questionnaire items. In context of gender difference in knowledge on oral cancer, many previous studies revealed highly variable results (Rahman et al., 2013; Chowdhury et al., 2010; Leao et al., 2005; Powe and Finnie, 2004). It is difficult to justify why females revealed better knowledge, but this can be partially ascribed to being more careful and they study harder during their graduate and postgraduate studies.

It is a foregone conclusion that with higher level of educations (specialties) the knowledge is higher. In our study, the general dental practitioners scored the lowest knowledge compared to the other specialties. Although, it was expected that oral/maxillofacial surgeons/pathologist/medicine specialists would have had higher knowledge compared to others, the prosthodontists were the best in many key items. Again, this is difficult to explain at least in light of the cross-sectional design of our study. In a recent study on prosthodontists in Saudi Arabia, they reflected good knowledge about oral cancer (Alqutaibi et al., 2020).

In our study, higher knowledge was obtained by those working in public sectors or universities compared to the private sector, and in the central and western regions compared to other regions of the Saudi Arabia. Concerning the working sector, this is simply due to the continuous educating programs conducted in the public sectors and universities, and the shorter working hours there in contrast to the private sector. Regarding the region of work, it is well known that the central and western regions of the Saudi Arabia are well developed and have had long time since the health infrastructures were established there compared to the other regions which are less developed. Consequently, for the health practitioners to persist in the central and western regions areas, they have to be highly competitive.

Each study has its own drawbacks and positives. Among the drawbacks is being of a cross-sectional, questionnaire-based design, and it is well-known the inherent weakness such studies had in terms of the pyramid of evidence. It is highly encouraged to conduct a prospective study in which the knowledge is measure pre- and post-training workshop on the topic. Another drawback is the small sample size relative to the huge population framework (dental practitioners in Saudi Arabia), though we tried our best to increase the number through many reminders using the different social media and emails. Another limitation is related to the multiple comparisons conducted which inflate the possibility of false positive responses due to the multiple comparison bias, although this is an inherited feature of the questionnaire-based studies owing to the numerous items (outcomes) and numerous independent explanatory variables included. Nevertheless, the current study is exploratory (hypotheses-generating); that is to say any positive association (false or true) must be dealt as a hypothesis for further research, not to build on it a practice or policy. Accordingly, interpretation of the results must be cautious, away from being over-extrapolated. However, and up to our knowledge, this study is the first that assessed the knowledge of dental practitioners in Saudi Arabia complications and management of radiotherapy to the head and neck area.

To sum up, our results show highly variable knowledge of dental practitioners on complications and management of radiotherapy to the head and neck area; that knowledge seems to fluctuate considerably with gender, experience, work sector, working region and specialty. The relevant authorities have the responsibility of provisioning and disseminating guidelines for proper approach in management of patients who are to undergo radiotherapy owing to head and neck cancer. Further, well-designed, large-scaled studies in this context are highly encouraged.

## Author Contribution Statement

The authors confirm contribution to the paper as follows: Study conception and design: Ahmed Shaher Alqahtani and Esam Halboub; data collection: Yaser Alhazmi, Yousef Alshamrani; analysis and interpretation of results: Esam Halboub; draft manuscript preparation: Ahmed shaher Alqahtani and Esam Halboub. All authors reviewed the results and approved the final version of the manuscript. 
